# Beyond Rest: Exploring the Sleep-Exercise Connection

**DOI:** 10.1192/j.eurpsy.2024.1603

**Published:** 2024-08-27

**Authors:** J. Kim, T. Kainth, E. Garrels, K. Tran

**Affiliations:** ^1^Psychiatry; ^2^Bronxcare Health System, Bronx, United States

## Abstract

**Introduction:**

The bidirectional relationship between the effects of sleep and exercise is often underappreciated. We aim to explore the bidirectional relationship of sleep and exercise. We further discuss the prominence of poor sleep in both the athletic and general population and understand the underlying mechanisms of interdependencies between the two. The goal is to illuminate practical implications to improve both areas and optimize physical and mental health.

**Objectives:**

- To explore the bidirectional relationship between sleep and exercise
- To understand how exercise can counterbalance the adverse metabolic consequences of sleep deprivation.

**Methods:**

We conducted a systemic literature review from Pubmed, Scopus, and PsychINFO using the search terms: “(exercise) and (sleep),” “(exercise performance) and (sleep),” “(sleep quality) and (exercise).” We included original studies in English conducted on age groups 18 years and older.

**Results:**

Data from 31 studies shows that a significant number of athletes experience poor sleep quality and daytime sleepiness. 68.5% of Qatar Stars League soccer players and 61% of collegiate athletes in NCAA institutions report daytime fatigue several times a week. Most common causes include overtraining, hectic travel schedules, and sleeping in unfamiliar settings. Studies confirm athletes often sleep less before intense training or competitions. Sleep deficiency may lead to reduced muscular strength and endurance, mood changes, increased perceived effort, impaired cognitive processing, and diminished motor skills. Athletes averaging less than 8 hours of sleep nightly were 1.7 times more prone to injuries. Physiologically, sleep loss alters ventilation, plasma lactate concentration, hormone secretion, and inflammatory responses, hinders muscle glycogen restoration. Extended sleep restriction decreases testosterone levels, which influence muscle mass, energy, bone strength, and more. On the contrary, exercise may counter adverse metabolic impacts of sleep deprivation. High-intensity interval exercise (HIIE) has shown to nullify negative metabolic effects of sleep deprivation, suggesting exercise’s protective potential.

**Conclusions:**

Sleep and exercise are fundamental to maintaining physical, mental, emotional, and spiritual health. The bidirectional, interdependent relationship can be best utilized by the providers to optimize overall well being. The critical impact of adequate sleep, particularly among athletes, is frequently underestimated. Poor sleep can detrimentally affect performance, amplify injury risks, and disrupt physiological functions, yet contemporary lifestyles often downplay its significance. It is important for healthcare professionals to emphasize a balanced approach to optimize these vital aspects. Continued research can offer strategies that benefit athletes and the broader populace, aiming to uplift daily life functionality.

**Disclosure of Interest:**

None Declared

